# Diagnosis and management of a mediastinal ectopic thyroid laying on the right bronchus: case report and review of literature

**DOI:** 10.1186/s12893-018-0354-y

**Published:** 2018-04-04

**Authors:** Alessio Metere, Tiziano De Giacomo, Massimo Vergine, Marco Biffoni, Laura Giacomelli

**Affiliations:** 1 Department of Surgical Sciences, Umberto I Hospital, “Sapienza” University of Rome, viale Regina Elena 324, Rome, 00161 Italy; 2 General Surgery, Surgical Specialties and Organ Transplantation “Paride Stefanini”, Umberto I Hospital, “Sapienza” University of Rome, viale Regina Elena 324, Rome, 00161 Italy

**Keywords:** Ectopic thyroid, Mediastinal thyroid, Mediastinal mass

## Abstract

**Background:**

The mediastinal ectopic thyroid is very rare, accounting for less than 1% of all cases of ectopic thyroid tissue. The differential diagnoses with other diseases such as lymphomas, thymic tumors and dermoid cysts is mandatory, in fact each one, needs different management and treatment.

**Case presentation:**

Here, we discuss a rare case of mediastinal ectopic thyroid presenting with a paratracheal mass laying on the right bronchus without symptoms. A 63-year-old male presented with an abnormal well-defined mass along the right paratracheal side, detected by chest x-ray. The CT scan confirmed the presence of a 6 × 8 cm heterogeneously enhanced mass, located behind the superior vena cava and left brachiocephalic artery, reaching azygos vein and right bronchus, without a mass effect. Taking into account the clinical importance of a mediastinal mass, we removed it surgically, through a double surgical approach consisting in a classical transverse cervicotomy for the left thyroid lobe, followed then by a longitudinal sternal splitting to remove the mediastinal mass and complete the thyroidectomy.

**Conclusions:**

In case of mediastinal masses, the surgical excision is recommended, presenting the double advantage to clarify the diagnosis and to treat the pathology. As demonstrated in this case, a mediastinal ectopic thyroid should be taken into account in the differential diagnosis, considering its clinical importance.

## Background

Thyroid gland is usually in the neck, anterolaterally from the second to fourth tracheal cartilages, less frequently could be found in other sites and it is called Ectopic Thyroid Tissue (ETT). The wall of the thyroglossal duct cyst is a common site for ETT, even if, other rare sites, such as retroperitoneum, adrenal gland, skull and lung have also been described [1–4]. The embryologic development gives reason to the main localization of ETT. The lingual thyroid tissue (at the base or below the tongue), accounts for 90% of these abnormalities followed by intratracheal ETT, while the mediastinal ETT is very rare, accounting for less than 1% of all cases [5]. The differential diagnosis of anterior mediastinal mass is very important and includes some pathologies such as lymphomas, thymic tumors, dermoid cysts and mediastinal ectopic thyroid which need different management and treatment. Here, we discuss a rare case of mediastinal ectopic thyroid presenting with a paratracheal mass on the right bronchus, without any symptoms.

## Case presentation

**Fig. 1:**
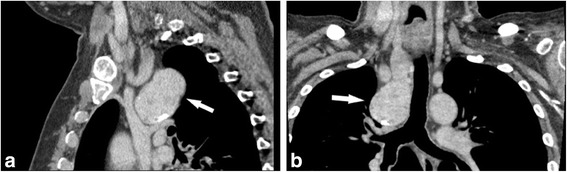
**a** Lateral view and **b** Frontal view of enhanced-CT scan of neck and chest showing the mediastinal mass (white arrow) and the mediastinal structures around

**Fig. 2:**
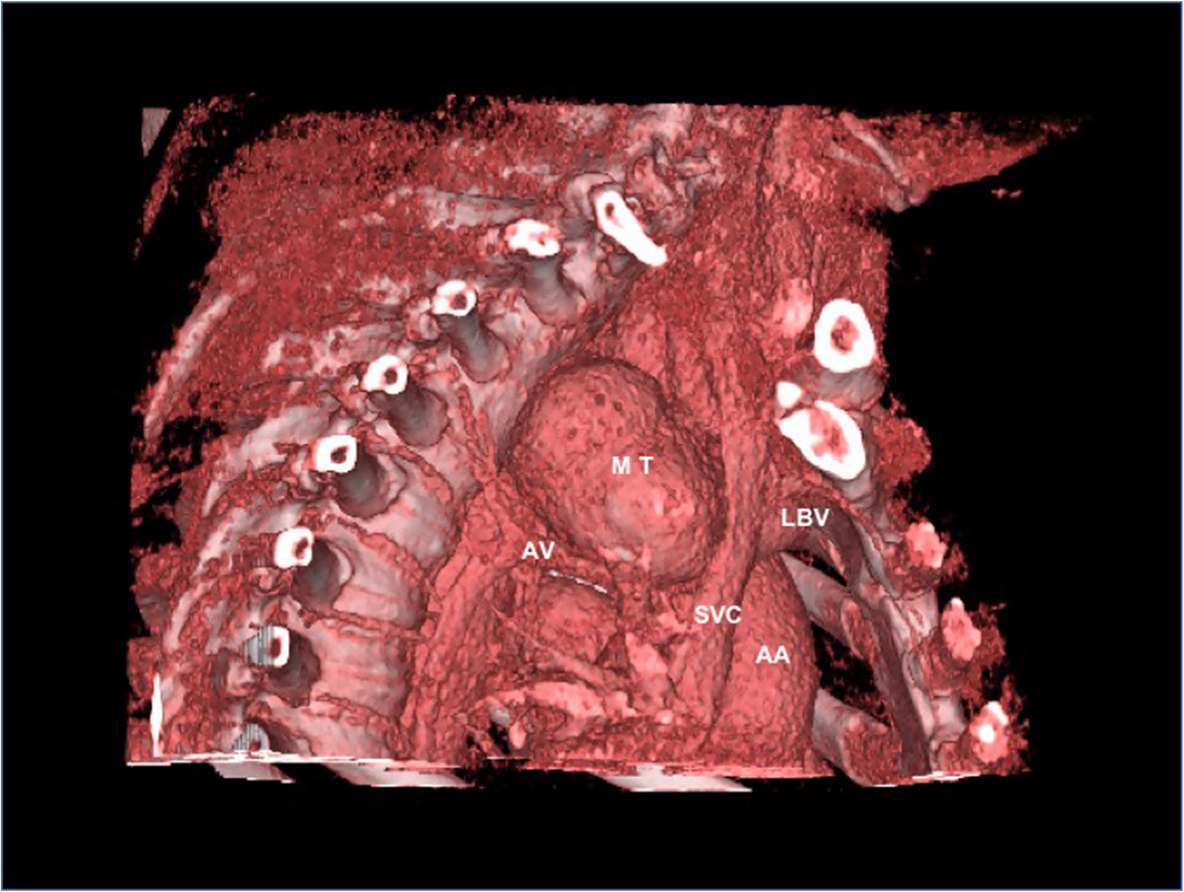
Tridimensional contrast Enhanced-CT reconstruction (AV: azygos vein; MT: mediastinal mass; SVC: superior vena cava; AA: ascending aorta; LBV: left brachiocephalic vein)

**Fig. 3:**
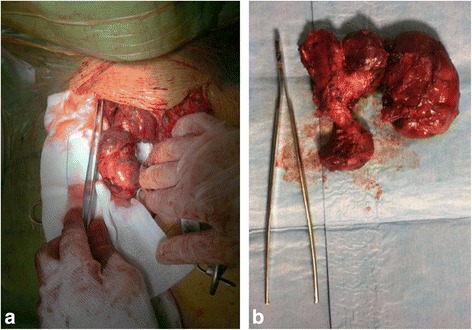
**a** Intraoperative isolation of mediastinal thyroid through sternal splitting. **b** Cervical and mediastinal thyroid after excision

A 63-year-old male presented with an abnormal well-defined mass along the right paratracheal side, detected by a chest x ray performed 2 weeks earlier, to exclude a diagnosis of pneumonia. We admitted the patient to our hospital to take the medical history, perform blood exams and contrast-enhanced CT scan, to better characterize the lesion. The CT scan of neck and chest confirmed the presence of a 6 × 8 cm heterogeneously enhanced mass in the right paratracheal area, characterized by some signs of necrosis inside. In particular, the mass was located behind the superior vena cava and left brachiocephalic artery, along the right side of the trachea, until reaching the azygos vein and the right bronchus, without any sign of compression regarding the organs around (Fig. 1). Interestingly, the contrast-enhanced CT scan showed a thin strip of tissue that linked the left thyroid lobe to chest mass, consisting with the hypothesis of a right “mediastinal thyroid lobe” (Fig. 2). No family history of thyroid disorder nor clinical signs of thyroid disease were detected. The results of his laboratory tests were all within normal limits and included a TSH level of 1.46 μUI/L (normal range, 0.40 to 4.00), free T3 of 1280 pmol/L (normal range, 600 to 1950), and free T4 of 12.3 pmol/L (normal range, 7.8 to 19.4). The serum levels of thyroid autoantibodies were also within normal ranges; Ab anti-TPO was < 25 U/mL (normal range, < 100) and Ab anti-TG was < 25 U/mL (normal range, < 100). As previously described, the differential diagnosis of anterior mediastinal mass is very important and includes some pathologies which need different management and treatment. We decided that the best treatment was to remove it surgically. We planned a double surgical approach consisting in a classical transverse cervicotomy to perform the left thyroidectomy followed by a longitudinal sternal splitting to remove the mediastinal mass and complete the thyroidectomy (Fig. 3). We preferred to perform a mini invasive surgery for the chest, through an upper partial sternal split, extended to the 4th intercostal spaces. Briefly, the manubrium was completely divided and the sternotomy carried to the level of the fourth interspace. The subsequent divarication of the sternum cause a fracture which then was later stabilized with sternal stitches. This technique was able to provide an effective surgical field, minimizing the surgical complication related to the total sternotomy.

**Table 1:** Literature Review Summary Table

Year	Author	Country	Title	Conclusions
2016	Cao L, et al. [1]	China	Clinical Characteristic and molecular pathology of skull ectopic thyroid cancer	The ETT was correlated with Akt/mTOR pathway high expression; orthotopic thyroid was related with MAPK/BRAF/ERK signaling pathway high expression; and the metastatic thyroid cancer was related with NFkB/MMP9 high expression.
2013	Ko HH, et al. [2]	Korea	Ectopic intrapulmonary thyroid: a case report	In this case the patient was previously operated for thyroid cancer and later an ectopic intrapulmonary thyroid was discovered at the follow-up.
2013	Wang SC, et al. [3]	Taiwan	Ectopic Thyroid Tissue in the Adrenal Gland Mimicking a Pheochromocytoma	Ectopic thyroid tissue occurring in the adrenal gland is extremely rare. Tsujimura t al. [11] reported the first case of ETTAG in 1996.2 Until now, there have been only 11 cases reported in the literature.
2017	Tamaki S, et al. [4]	Japan	Laparoscopic resection of retroperitoneal ectopic thyroid tissue	This case shows the unusual finding of ectopic thyroid tissue in the retroperitoneum and the usefulness of laparoscopy for resecting such masses.
2003	Gamblin TC, et al. [5]	USA	Ectopic thyroid	Here is presented a case of ETT extended from the thoracic inlet inferiorly, anterior to the heart, with severe tracheal deviation to the patient’s right.
2004	De Felice M, et al. [6]	Italy	Thyroid development and its disorders: genetics and molecular mechanisms	Gene-targeting experiments have demonstrated that Foxe1 is required for thyroid migration and that mice homozygous for Foxe1 mutations show a sublingual thyroid.
2013	Nettore IC, et al. [7]	Italy	The molecular causes of thyroid dysgenesis: a systematic review	Several mutations in genes playing a role during thyroid morphogenesis such as NKX2-1, PAX8, FOXE1, NKX2-5, TSHR, have been reported in ETT
2010	Abu-Khudir R, et al. [8]	Canada	Transcriptome, methylome and genomic variations analysis of ectopic thyroid glands	This study analyzed gene expression, genome-wide methylation, and structural genome variations in normal versus ETT, demonstrating a differential gene expression in ETT.

The histological exam confirmed that the chest mass was thyroid tissue, no neoplastic cells were found but only the typical features of the multinodular goiter. No post-operative complications were observed, neither hypocalcemia nor nerves injuries, thus the patient was discharged on the sixth post-operative day.

## Discussion and conclusions

As previously described, the wall of a thyroglossal duct cyst is the common site for ETT followed by others considered very rare. The most common and reasonable hypothesis to explain the presence of ectopic thyroid, takes into account some defects in the differentiation, migration or growth of thyroid tissue, happened during the embryological development. However, other interesting working hypothesis takes into account the role played by genetic expression in the understanding of molecular mechanisms regulating the size, shape, and position of thyroid [6]. In these cases, in fact, several mutations in genes playing a role during thyroid morphogenesis such as NKX2-1, PAX8, FOXE1, NKX2-5, TSHR, have been reported [7]. Abu-Khudir R. et al. analyzed gene expression, genome-wide methylation, and structural genome variations in normal versus ETT, demonstrating a differential gene expression in ETT [8]. Unfortunately is necessary a large cohort to reveal and confirm, definitively, the involvement of one or more genes as responsible for defective thyroid migration during embryogenesis. It is important to underline that the mediastinum contains several pluripotent cells, that justify the large variety of tumors, such as lymphomas, endocrine or neuroendocrine carcinomas, primary thymic carcinomas or mesenchymal tumors, that can be found in this anatomical district. Hodgkin lymphoma, large B cell lymphoma and lymphoblastic lymphoma are the most common mediastinal lymphomas, while thymic and neuroendocrine carcinomas are rarer but highly malignant. Moreover, lymphomas and neuroendocrine carcinomas are usually located in the anterior mediastinum while the neurogenic tumors are frequently found in the posterior side. The localization in the mediastinum of mesenchymal tumors is very rare and usually uneventfully [9] (see table 1 for literature review). Mediastinal goiter is usually asymptomatic and consequently is discovered incidentally, like in our case, until the structures in the chest remain uninvolved. On the contrary, the patients refer dyspnea, cough and a sensation of retrosternal mass, often in case of mediastinal thyroid carcinomas. It is possible to clarify the diagnosis using several approaches, such as fine needle aspiration (US or CT-guided) or endobronchial ultrasound-guided transbronchial needle aspiration [10]. The mentioned techniques help us obtain information about the nature of the mass, even if they do not usually influence the surgical procedure. We discussed about the opportunity to perform a biopsy before surgery, but we decided to avoid it because of the high risk of complications due to the several anatomical structures surrounding the mass. Moreover, considering the anatomical dislocation of the mass, from the neck to the right bronchus, the opportunity to obtain histological samples representative of the entire tissue would have been very rare. In fact, for mediastinal mass and in particular for mediastinal thyroid mass, surgery should be considered because of the high risk of compression of the surrounding mediastinal organs. In conclusion, according to our experience, the surgical excision of mediastinal ectopic thyroid is recommended to obtain, at the same time, the definitive diagnosis and treatment. Frequently, it represents the elective treatment in case of thyroid cancer or multinodular goiter, taking into account that both tend to increase their mass and compress the mediastinal structures around.

## Abbreviation

ETT: Ectopic thyroid tissue
